# A Virtual Fracture Clinic Pathway for Managing Suspected Paediatric Scaphoid Fractures

**DOI:** 10.7759/cureus.29238

**Published:** 2022-09-16

**Authors:** Karim Aboelmagd, Tariq Aboelmagd, Jennifer C Lane, John Morley, Claire Middleton, Amr El Khouly, Neville Davies

**Affiliations:** 1 Trauma and Orthopaedics, Royal Berkshire Hospital, Reading, GBR; 2 Trauma and Orthopaedics, Nuffield Orthopaedic Centre, Oxford, GBR; 3 Truama and Orthopaedics, Royal Berkshire Hospital, Reading, GBR

**Keywords:** service provision, paediatric scaphoid fracture, occult scaphoid fracture, virtual fracture clinic, delayed scaphoid fracture diagnosis

## Abstract

Introduction: The mismanagement of an occult scaphoid fracture is a significant concern in patients presenting with anatomical snuffbox tenderness and no radiographic signs of injury.

Aim: This study investigated whether a virtual fracture clinic (VFC) could improve care efficiency and expedite management decisions surrounding suspected pediatric scaphoid fractures.

Method: Data was reviewed for patients referred via the VFC for suspected scaphoid fractures at a local trauma unit over 19 months. Patients received an "appointment" in VFC. Based on their notes and imaging, patients were referred to an outpatient clinic for repeat radiographs within two weeks (if initial radiographs demonstrated no fracture). Patients with unremarkable second x-rays were contacted and informed to mobilize and return if the pain persisted at four weeks.

Results: The pathway received 175 referrals; 114 male, 61 female, mean age 14 years, range 9-17) with 42 scaphoid fractures diagnosed, 35 (83.3%) on first x-ray, and 7 (16.7%) occult fractures. The pathway managed all patients as intended; 71 patients were seen face-to-face in the clinic due to age or pathology picked up on the first x-ray, and 104 required repeat radiographs. Following the second radiograph, 78 patients were discharged directly. Twenty-six patients required further review in a face-to-face clinic after their second radiograph.

Conclusion: VFC appears to be a safe and efficient method of managing patients with suspected scaphoid fractures on short-term follow-up analysis. This cohort presents no 'missed' injuries and therefore appears safe compared to conventional treatment pathways.

## Introduction

Scaphoid fractures are the most common carpal bone fracture in the pediatric population, with an average annual incidence of 11 per 100,000 [1]. They account for 0.34% of all pediatric fractures, 0.45% of all pediatric upper limb fractures, and 3% of all pediatric hand and wrist fractures [2-5]. Scaphoid fractures can be challenging to diagnose at initial presentation due to the lack of acute radiological signs in 30% of cases [6]. Although rare, missed scaphoid fractures may lead to non-union, avascular necrosis, and significant morbidity; previous work suggests that injury characteristics in children mirror that seen in adults [5,7,8].

 Traditional outpatient fracture clinic models review the patient a week or more post-injury with clinical assessment and repeat designated scaphoid radiographs [9,10]. Unnecessary clinic visits can be time-consuming for clinicians and parents (who often have to take time off work) and upsetting for young children. The unrestrained ordering of advanced 3D imaging on all pediatric patients with potential scaphoid injuries is impracticable and expensive. To improve service provision across the National Health Service (NHS), the British Orthopaedic Association produced guidelines regarding the follow-up of closed fractures in an outpatient setting [11]. Streamlining the number of visits a child undertakes following injury is of increased importance to prevent distress for the child and caregiver and reduce the impact of repeated time away from school and leisure activities.

To address these issues, some trauma and orthopedic surgery departments have successfully established virtual fracture clinics (VFC) to improve patient care efficiency [12-14]. This new model, with early senior triaging using clinical notes and radiographs from the initial presentation, allows for a targeted management plan to be relayed to the patient via telephone the same day. This model aims to streamline the patient management pathway, avoid unnecessary appointments, and see the most appropriate clinician when arriving at the clinic. VFC initiatives have reduced waiting times, improved patient satisfaction, and reduced costs in adult services without increasing consultation times, primarily when covering a large geographical area [12-14]. They are currently used across the UK for triaging most orthopedic referrals that do not require urgent attention by the on-call orthopedic team.

The success of VFC pathways locally in managing adult orthopedic injuries has led to an interest in establishing a similar service for pediatric orthopedic injuries. Many pediatric patients are in the unit; some travel from a wide geographical area. Improving patient flow through busy clinics and avoiding unnecessary anxiety from repeated hospital visits and time away from school is pertinent. Local patient feedback had suggested that fewer consultations were of importance to families. Considering the heightened concern surrounding missed pediatric injuries, especially in an injury like suspected scaphoid fractures where misdiagnosis has significant consequences, a study into a VFC system's safety for pediatric patients was warranted.

This study aimed to determine if a VFC pathway could safely manage patients with suspected scaphoid fractures.

## Materials and methods

Methods

We conducted this quality improvement project in one urban district general hospital, with a catchment population of 490,000, of which 20% are under 14. This hospital is the 'hub' of a hub and spoke model for surrounding rural minor injury units (MIUs) led by nurse specialists without surgeons on site. Minor injury units are small units in more peripheral locations dealing with injuries that need swift attention but are not critical or life-threatening, such as strains, cuts, non-limb-threatening fractures, minor burns/scalds, etc. A Multidisciplinary team designed and introduced a VFC pathway to the department.

Intervention: Virtual fracture clinic pathway

Confirmed or suspected scaphoid fractures in all patients under 18 years were immobilized in either a removable wrist splint (with Velcro strap) or below elbow scaphoid cast. Any open or bilateral injuries required direct referral to the on-call orthopedic team. The patient details and Emergency Department (ED) clerking were sent electronically to the orthopedic department for VFC review. Patients were provided an address and two contact phone numbers when registering in ED to provide multiple modalities of contact. Contact details of the fracture outpatient clinic were given to all patients before leaving ED to allow them to contact our team should they be missed.

The on-call orthopedic consultant reviewed the clinical information and radiographs in the VFC. The family was contacted if the radiographs suggested a scaphoid fracture, if the history and radiographs suggested a different injury or if the patient was under 12 years. The patient was seen face to face in the next pediatric outpatient fracture clinic (Table 1). VFC documentation was made available to the clinician seeing the patient in the clinic. The VFC team consisted of the on-call consultant who reviewed the images and history, a nurse who contacted patients and informed them of ongoing management plans, and an administrator who organized the referrals and documented decisions. 

**Table 1:** Indication for direct face-to-face clinic appointment following initial presentation to ED

Indications for Initial Face to Face clinic
Initial radiograph demonstrating a scaphoid fracture
Age under 12 years
History or radiograph suggesting an injury other than that of the scaphoid
History inconsistent with radiographic findings

For those that did not meet the criteria for a direct face-to-face appointment, the family was contacted and provided with an open appointment to attend their local radiology department in 10 - 14 days from the date of injury for repeat scaphoid radiographs. The VFC reviewed the repeat radiographs the following working day. A senior nurse contacted the family by phone if no fracture was identified and advised the patient to remove the splint and mobilize the wrist. Written confirmation was sent to all families via post. Families could request a face-to-face appointment if no bony injury was seen on repeat radiographs. If a patient had persistent pain four weeks after this phone call, they were advised to contact the orthopedic department, and a face-to-face fracture clinic review was organized. A physician would review the patient in this clinic and arrange other 3D imaginings if appropriate. Patients with pathology noted on repeat radiographs at 10-14 days were seen in the next pediatric outpatient fracture clinic.

**Figure 1:**
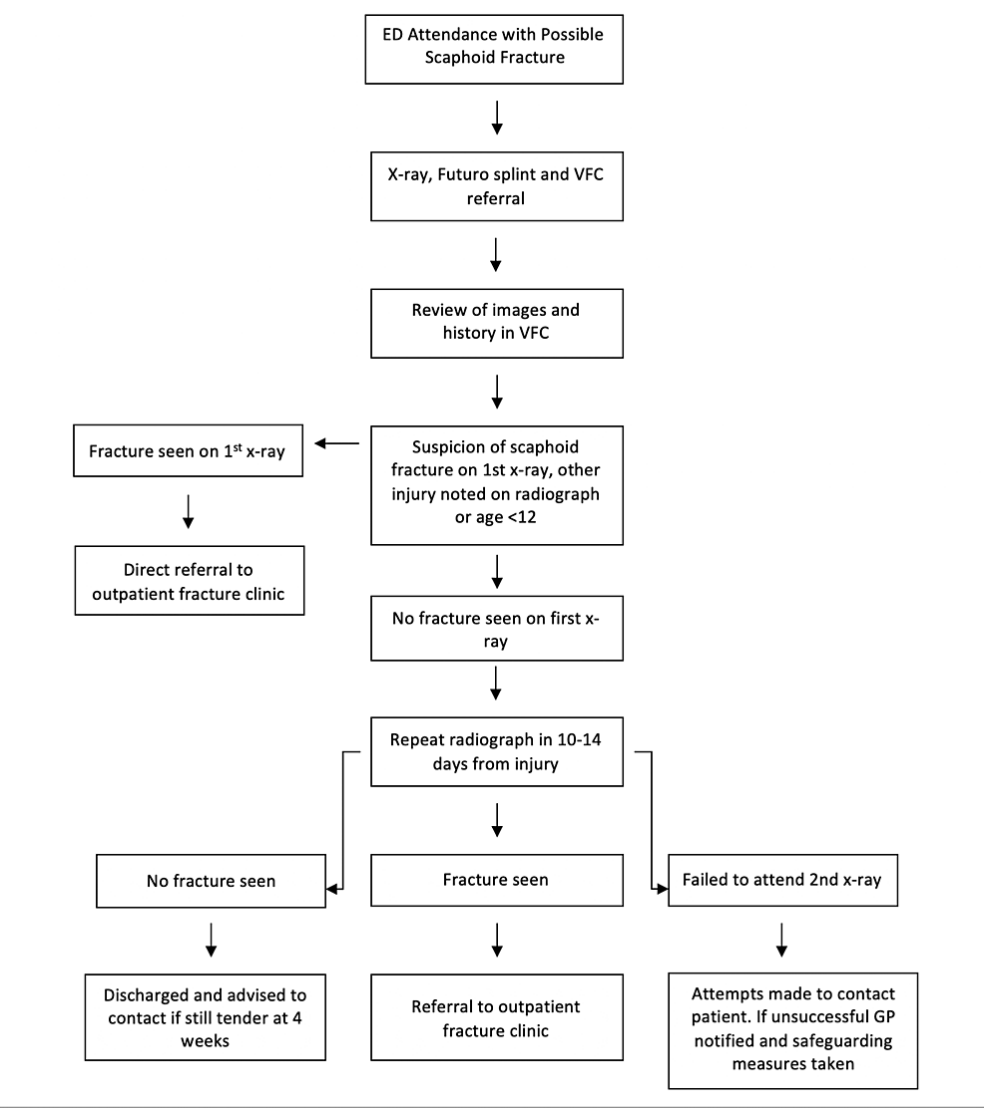
Paediatric VFC pathway flowchart

The VFC team made all attempts to contact the patients via telephone with an ongoing plan on the same day the consultant had reviewed their radiograph. (Figure 1). The on-call consultant reviewed all repeat 10-14 day radiographs the following working day.

VFC outcomes were relayed to patients via phone and post, with multiple attempts made if the first contact was unsuccessful. If the family did not respond to calls, voicemails and messages were left with instructions to call the VFC. If families did not engage with the pathway or respond, safeguarding measures were followed. Their family doctor is contacted (in the UK, the general practitioner is the gatekeeper to care and facilitates lifelong healthcare provision, including social care needs). 

Study design

Electronic patient records and imaging were used to undertake a retrospective review. All the pediatric patients with a suspected scaphoid fracture from the initiation of the VFC pathway in August 2015 to March 2017 were included. Data collected included; details of the injury, presentation location, VFC assessment, presentation for follow-up imaging, management plan, and any re-presentation to medical services or complications. Outcomes collected for quality improvement were the number of appointments undertaken per patient and the number of patients compliant within the pathway. Outcomes collected for safety were the number of patients representing with ongoing symptoms or problems, including those with missed injuries or complications. A descriptive analysis was undertaken. Where numbers are below 5, numbers are suppressed to prevent secondary disclosure of data. 

Ethical approval

This study was registered within the hospital trust, N5104.

## Results

Patient demographics and mechanism of injury

The service received 175 referrals over 19 months for confirmed or suspected pediatric scaphoid fractures. The age range was 9-17 (mean 13.8, SD 2.3). Of those referred, 35% (61) were female, and 65% (114) were male. Median 20 months follow up, Range 9.4 - 29.2 months. Forty-two scaphoid fractures were diagnosed with 71% (30) in male patients and 28% (12) in female patients. 83% (35) of scaphoid fractures were visible and diagnosed on initial radiographs. Repeat radiographs or further 3D imaging identified a further 16.7% (7). These included three waist fractures, two distal pole fractures, and two scaphoid tubercle fractures.

Table 2 gives details of the mechanism of injury. The most common mechanism of injury of all patients referred was a fall onto an outstretched hand (FOOSH) - 69% (121), followed by a fall off a bike (15%, 27 patients). 

**Table 2:** Mechanism of Injury for all paediatric patients referred to VFC with a suspected scaphoid fracture (<5 to prevent secondary disclosure of data for included patients)

Mechanism of Injury	Frequency (%)
FOOSH	121 (69)
Fall off bike	27 (15)
Direct blow to wrist	11 (6)
Forced Wrist Extension	<5
Punch	6 (3)
Road Traffic Collision	<5
Forced Wrist Flexion	<5
Fall Secondary to Seizure	<5
Unknown	<5
	175

Of the 42 scaphoid fractures identified, the most common injury mechanism was a FOOSH (64%) followed by a fall off a bike, representing 24% of fractures identified (Table 3).

**Table 3:** Mechanism of Injury of confirmed paediatric scaphoid fractures (<5 to prevent secondary disclosure of data for included patients)

Mechanism of Injury	Identified on First Radiograph (%)	Identified on Further Imaging	Total
FOOSH	23 (66%)	4 (57%)	27
RTC	<5	0	<5
Punch	<5	0	<5
Hit by an object	<5	0	<5
Fall off bike	7 (20%)	3 (43%)	10
	35	7	42

No patients with occult fractures required surgery. Of the 35 patients diagnosed on initial imaging, 6 underwent surgery. Percutaneous fixation was used to manage 1 Proximal pole fracture and two waist fractures. Open reduction and Internal fixation with bone grafting were performed in 3 patients with scaphoid waist fractures. No patient underwent multiple procedures. 

Compliance with the pathway

**Figure 2:**
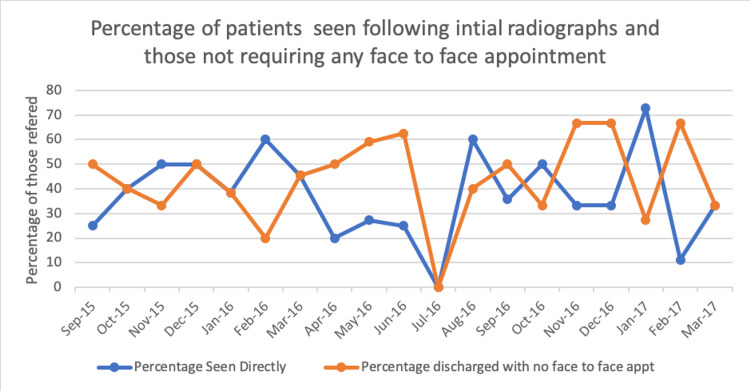
Run chart demonstrating the percentage of patients referred to the VFC who required a face to face consultation after their initial radiographs, as well as the percentage of patients who were discharged from the clinic without a face-to-face appointment following a repeat radiographs

Initial radiographs identified 41 patients with pathology who were seen in the face-to-face clinic. Of the remaining 134 patients left, 30 were seen directly in a face-to-face fracture clinic before having repeat radiographs, with 104 patients managed as the pathway intended (Figure 2).

The families of 101 of the 104 patients requiring repeat radiographs at 10-14 days (97%) were contactable by phone. One family was referred through the hospital child safeguarding process as they were uncontactable via the details they had provided.

Safety

Following 2nd X-rays, 78 patients were discharged without a face-to-face appointment. Twenty-six patients required further review in a face-to-face outpatient clinic after their second radiograph. Of these 26, 19% (5) seen in the clinic underwent an MRI, and 19% (5) underwent a CT scan of the wrist. Three of the 10 patients who underwent 3D imaging were diagnosed with fractures (1 distal radius fracture and two distal pole fractures).

The average time from presentation to ED to consultant review of a radiograph was 1.56 days (range of 1-7 days). Two patients presented a second time through the open clinic policy, with pain at a mean of 6 weeks (range 4-8 weeks). This safety net of returning to the clinic allowed examination and further investigation through 3D imaging. Both patients were subsequently discharged, with no fracture or pathology found. 

## Discussion

This study investigates if a VFC pathway can be used to manage suspected pediatric scaphoid fractures safely. Over the 19-month study period, we found high compliance with the pathway from patients and families. Deviation from the pathway was primarily due to nurse specialists in surrounding clinics placing children in casts at the initial presentation. We had two re-presentations within the time limit of this study. Both patients were subsequently discharged pain-free after 3D imaging showed no bony injury. No patient was discharged with continued pain, and no occult fracture required surgical intervention.

The pathway complies with the British Orthopaedics Associations Standards for Trauma regarding fracture clinic services. A consultant orthopedic surgeon reviewed all patients' imaging, history, and clinical examination findings within 72 hours of the presentation of injury by a consultant orthopedic surgeon. The pediatric orthopedic consultant-led clinic reviewed all patients requiring face-to-face consultation. All patients had rapid access back to a pediatric fracture clinic if required. 

Our cohort of 42 pediatric patients with a confirmed scaphoid fracture demonstrated a male predominance (M: F ratio of 2.5:1), keeping with the already published literature [1,2,4,5]. The most common mechanism of injury in our cohort for patients presenting with a suspected scaphoid fracture and patients with a confirmed scaphoid fracture was a fall onto an outstretched hand, representing 69% and 64%, respectively. Gajdobranski et al. found a similar cohort in their study [4]. 

By allowing patients to present to any of the three minor injury units within our catchment area between 10-14 days post-injury for the second set of radiographs, we aimed to limit the time required away from school and also eased parental requirements to take time off work to attend a fixed radiology or clinic appointment. Face-to-face consultations were not required for 75% of patients referred via the VFC stream, releasing these appointments for other patients and positively impacting efficiency.

In total, the pediatric outpatient clinic saved 78 outpatient appointments, costing £8,814 ($11,443). The traditional pathway for managing scaphoid fractures involves cast immobilization followed by clinic review and repeat radiographs at two weeks. Studies have demonstrated that early/immediate 3D imaging is cost-effective in managing suspected scaphoid fractures compared to the traditional pathway [15-17]. No studies were identified comparing immediate 3D imaging to a virtual pathway for managing a suspect's scaphoid fractures. The use of early MRI in diagnosing occult fractures within the NHS has been proven cost-effective [17]. Rue et al. showed significant cost-effectiveness of immediate MRI in managing suspected occult scaphoid fractures; however, this study only included patients presenting Monday to Friday between 07:30 and 18:00 in patients over 16 years of age. In centers with more limited access to MRI, we believe this pathway represents an acceptable alternative. 

Unfortunately, 30 patients required direct consultation and could not participate in the VFC pathway as anticipated. There were two main reasons for this. Firstly clinicians at our peripheral MIU were unfamiliar with the protocol and directly booked patients into fracture clinics. Secondly, those patients placed in an entire scaphoid cast required cast removal before attending their second radiographs. This finding was a good learning point to arise from this study. We have subsequently re-educated all clinicians seeing these patients in our ED and peripheral MIU units and advised, where possible, to use removable wrist splints instead of full scaphoid casts in the first instance to improve the uniformity of care for all patients in the region. 

Limitations

This pathway is not without limitations. Entry to the pathway relies upon the correct initial assessment of non-orthopedic specialists examining pediatric patients with an acutely painful wrist and a normal radiograph. There is a risk that patients could be unnecessarily imaged, or other non bony injuries could be missed by not clinically reviewing patients prior to repeat imaging. The use of repeat radiographs to rule out scaphoid fractures is still common practice in UK hospitals. However, they are unreliable compared with MRI and CT and demonstrate poor inter-observer agreement [18-20]. In the progressively ossifying scaphoid of the pediatric patient, the plain film diagnosis of scaphoid fractures can be made more challenging by fractures that may occur through the osteochondral interface. We may have missed undisplaced scaphoid fractures that have gone on to unite because they were stable and remained undisplaced at two weeks. We cannot state that no fractures were missed as most patients did not receive the gold standard imaging model.

To assess the long-term safety of the pathway, extended follow-up is required. No patients have presented with missed pathology on short-term follow-up. The study was undertaken in all centers to limit bias of only patients presenting to the hub. This modification emphasized the need for further education on the pathway design in the spoke centers.

## Conclusions

Based on short-term follow-up, the VFC management of pediatric scaphoid fractures appears to be a safe and efficient method of managing children with suspected scaphoid fractures. It allows for expedited senior review and increased efficiency of referrals across a wide geographical area at significantly reduced costs compared to conventional treatment pathways. The pathway had benefits for the general orthopedic fracture clinics. The pathway continues in the department, sustained using the established VFC system for adult patients. Further work is now focused on using VFC pathways for other pediatric orthopedic injuries and ensuring uniformity of care across all referring sites.
